# A qualitative investigation of the impact of peer to peer online support for women living with Polycystic Ovary Syndrome

**DOI:** 10.1186/1472-6874-13-51

**Published:** 2013-12-17

**Authors:** Sarah Holbrey, Neil S Coulson

**Affiliations:** 1 School of Health Studies, Horton A Building, University of Bradford, Richmond Road, Bradford BD7 1DP, UK; 2 Division of Rehabilitation & Ageing, The Medical School, B floor, Queen’s Medical centre, University of Nottingham, Nottingham NG7 2UH, UK

**Keywords:** Polycystic Ovary Syndrome, PCOS, Chronic conditions, Online support, Peer support

## Abstract

**Background:**

Polycystic Ovary Syndrome is a common, chronic condition which affects women living with the condition both physically and psychologically. Social support may be beneficial to sufferers in coping with chronic conditions and the Internet is becoming a common place for accessing social support and information. The aim of this study was to consider the experiences of women living with Polycystic Ovary Syndrome who access and participate in an online support group discussion forum dedicated to issues surrounding this condition.

**Methods:**

Fifty participants responded to a series of open-ended questions via an online survey.

**Results:**

Thematic analysis revealed a number of empowering and disempowering experiences associated with online support group participation. The empowering processes reported by members of the group included: Connecting with others who understand; Access to information and advice; Interaction with healthcare professionals; Treatment-related decision making; Improved adjustment and management. In terms disempowering processes, only two were described by group participants: Reading about the negative experiences of others and Feeling like an outsider.

**Conclusions:**

For women living with Polycystic Ovary Syndrome, participation within an online support group may help to empower them in a range of important ways however, there may be some disempowering consequences.

## Background

Polycystic Ovary Syndrome (PCOS) is a chronic disorder affecting women of reproductive age, diagnosed by the presence of at least two of the following: the presence of numerous, small ovarian cysts; menstrual cycle disruptions; or clinical signs of hyperandrogenism [1]. Some women may present with polycystic ovaries but have no other symptoms, in which case they are referred to as having polycystic ovaries without the syndrome [2]. Hyperandrogenism is a hormonal disturbance that can produce a variety of clinical manifestations in women, including fertility problems, weight gain/obesity, hirsutism, acne, alopecia, and mood changes [3]. Despite being the most common endocrinopathy disorder, believed to affect 6-10% of reproductive-age women, PCOS is the least understood and this may be attributable to the fact that PCOS is difficult to diagnose and symptoms vary in both presence and severity [4,5]. Although incurable, treatment may include lifestyle changes, as well as medication and surgery and whilst these options may help sufferers manage individual symptoms [6], they are often accompanied by a range of side effects [7]. Women with PCOS also have an increased risk of developing other health complications, including type 2 diabetes, cardiovascular disease, stroke and endometrial cancer [8]. Furthermore, PCOS sufferers may also have a higher risk of complications during pregnancy, particularly if they are also obese [9].

Psychological research has largely centred around the psychosocial impact of PCOS and it is perhaps unsurprising, given the scope of health problems and unpleasant physical manifestations of the condition, that PCOS and its related symptoms have been found to be associated with poorer quality of life and increased psychological distress in sufferers [10-12]. In one study using standardised measures of psychological distress, women with hirsutism showed higher scores for anxiety and depression than patients who had been newly diagnosed with gynaecological or breast cancers [13]. However, there exists some disagreement as to which symptoms produce the most negative impact in women with PCOS [8,14-16].

In terms of opportunities to improve quality of life in women with PCOS, Ching, Burke and Stuckey [17] found that a higher quality of life as assessed by the SF-36 was associated with a perception of better information being provided overall and in the areas of menstrual disturbance, hirsutism and long-term health specifically. The authors suggest that one route to improving the quality of life in women living with PCOS, may be through access to and provision of, more comprehensive and relevant information.

Support groups may be a useful means through which women living with PCOS may have access to more detailed and personally relevant information. Indeed, recent evidence suggests that for those women who participate in support groups, there may well be a beneficial effect in terms of minimising the negative psychosocial impact of PCOS and symptoms related to it, such as infertility [18,19]. Similarly, the Internet has also provided new opportunities for information and advice and it has been found to be helpful to women with PCOS in terms of shared decision making with health professionals [20]. Furthermore, it was regarded as a convenient, private and accessible means through which support could be obtained. However, researchers have concerns over the credibility of information available on PCOS-related websites [21].

In recent years, health-related online support groups (OSGs) have been shown to be an increasingly popular source of information, advice and support [22,23]. In particular, individuals living with chronic illness may view them as a safe place to share their concerns and to learn about the realities of living with a particular health problem [24]. Whilst there exists few randomised controlled trials [25] that have tested the efficacy of online support groups, there is a growing body of qualitative and cross-sectional literature that has described a range of potential benefits [26-28]. Indeed, recent work has adopted an empowerment framework through which to consider the potentially empowering processes that may underpin online interaction between patients [29-31]. Whilst the concept of empowerment has been widely debated within the literature, it is generally considered to be an active, participatory process through which individuals, organisations, and communities gain greater control, efficacy, and social justice [32]. Thus far, research studies have described a range of potentially empowering processes linked with the use of online support groups including: exchanging information, encountering emotional support, finding recognition, sharing experiences, helping others, and amusement [29-31]. Furthermore, a number of empowering outcomes have also been identified and include: being better informed, feeling confident in the relationship with their physician, their treatment, and their social environment; improved acceptance of the disease; increase optimism, and enhanced self-esteem and social well-being. However, it should be noted that such work has focused on a very narrow range of conditions such as arthritis, fibromyalgia, breast cancer and HIV/AIDS [29-31] and the ways through which online support participation may empower members of PCOS online support groups has not been the focus of any empirical study.

Online support groups are not without their potential difficulties and recent evidence has similarly described a number of ways through which participation may negatively impact upon members. For example, Coulson [33] reported that members of Inflammatory Bowel Disease online support groups experienced a number of difficulties with regards assessing the quality and credibility of information exchanged online, the negative representation of the illness online as well as interpersonal problems. Other studies have similarly reported an array of potential difficulties though these have varied according to the type of illness support group under investigation. Very few studies [29-31] have considered the disempowering effects of online support group participation, and in the context of PCOS this also remains an under researched issue.

In the context of PCOS, online support groups may be of particular benefit to teenagers with PCOS, as this group may require more peer-to-peer support compared with adult women [34]. However, only one study to date has explored the role of online support groups for women living with the condition [35]. This study analysed a sample of messages which had been posted to a PCOS-OSG and suggested a number of ways in which women may potentially feel supported and informed through participation. However, one limitation of this particular method is that it does not ask group members *directly* about their views and experiences of living with PCOS, so the extent to which they actually feel supported remains unclear. Therefore, the aim of the present study is to explore directly, using a qualitative method, the views and experiences of women about how, if at all, their participation in an online support group for PCOS may empower them as well as whether there exists any potentially disempowering processes arising from the online interactions.

## Methods

### Recruitment

Participants were recruited from a UK-based online support group, specifically for women affected by PCOS. Run by volunteers, the group is an active, registered charity which is closely involved in collaboration with research and education on PCOS. This group was selected on the basis that it was identified as one of the most active for women suffering with PCOS. With the aim of providing a space for peer support, discussion and information exchange, the group provides an active, peer-led discussion forum with over 4,600 registered members at the time this study was conducted, who had posted over 340,000 items between them on the forum. The group requires users to register as a member before having permission to submit posts, whilst non-members may still access the group to view submitted posts.

Following ethical approval from the Ethics Committee at the University of Nottingham, moderators of the group were contacted to seek permission to access members. An advertisement for volunteers to participate in the study was posted on the forum by the moderators on behalf of the researcher. This message requested women who used the group and who either had symptoms of PCOS or an official PCOS diagnosis, to voluntarily participate in the study. Information about the purpose of the study and a web-link to the online questionnaire was provided. In addition, emails from the moderators of the group were sent to 150 users from a quota of approximately 1,000 users who had previously registered to receive invitations to volunteer in research. Unfortunately, due to issues of confidentiality it was not possible for the researchers to be given access to the list of members’ emails. As a consequence, it was not possible for the researchers to randomly select members to receive the invitation to participate. Moreover, according to the moderators it was not possible to undertake a random sampling approach on our behalf and therefore the approach outlined above was the only means through which the study could proceed.

### Materials

Data were collected via an online questionnaire, which consisted of both closed and open-ended items. Closed items included the following: demographic information of age and gender; participants’ PCOS medical status such as confirmation of diagnosis and presence and severity of symptoms; participants’ use of OSG, such as duration of involvement, frequency of access and level of engagement with the group, in terms of whether or not participants posted messages on the forum, and the frequency of this. In addition, 7 open-ended items explored participants’ motives for accessing the online support group as well as their experiences thus far (see Table 1). These items were based on similar questions that have been used in other online qualitative studies exploring the experience of engaging with online support groups across a range of chronic conditions [24,36] and were approved by the group moderators.

**Table 1:** Open-ended questions included in the online survey

1.	What first led you to start using the online support group?
2.	Can you explain what your main motives are now in using the online support group – or are they still the same as when you first started?
3.	Do you feel there are any benefits to you in using the online support group, and if so, what are these?
4.	Do you feel there are any negative aspects in using the online support group, and if so, what are these?
5.	Can you explain if using the online support group has changed your perceptions and experience of living with PCOS/symptoms?
6.	How do you feel that using the online support group has changed the way you communicate with healthcare practitioners about your PCOS/symptoms, if at all?
7.	Do you have any further comments about your experiences within the online support group?

### Participants

A total of 64 participants responded to the study invitation. Informed consent was obtained from all participants as the survey did not allow participants to proceed without indicating this consent. Fourteen were excluded on the following basis: one indicated they were ‘male’, three stated that they were new and unable to respond to the questions, one indicated they did not use the OSG, one was a duplicate response, five did not answer any of the open-ended questions and a further three provided very limited responses. Fifty female participants were therefore included in the qualitative analysis, 48 of which had official PCOS diagnoses and two participants who were undiagnosed but experienced a range of symptoms consistent with PCOS. Participants ranged in age from 20 to 45 years (mean = 33.6 years; SD = 5.36). The majority (58%) of participants had been diagnosed with PCOS for at least 5 years; 22% for between 2–4 years; 10% for between 1–2 years and 6% for less than 1 year. Twenty-two percent of participants had used the OSG for at least 5 years; 36% for between 2–4 years; 22% for between 1–2 years; 6% for between 6 months to 1 year and a further 14% had used the OSG for less than 6 months. Approximately half (52%) of participants accessed the OSG only ‘occasionally’; 20% accessed it ‘weekly’ and 18% accessed it ‘daily’. In terms of their level of engagement with the OSG, 16% of participants stated that they ‘frequently’ posted messages; 26% stated they ‘sometimes’ posted; 34% of participants ‘rarely’ posted and 24% ‘never’ posted.

### Procedure

Clicking on the web-link provided in the advertisement for study volunteers led participants to the online survey, hosted via the online survey generator SurveyMonkey®. Participants were given further information about the study including their right to withdraw and were reassured that their responses would be kept anonymous. To proceed, all participants were required to indicate their consent to participating. Participants who gave consent were directed to the start of the questionnaire. Participants were also asked if they consented to direct quotes of their responses being used in the reporting of the research.

#### Analysis

Results from the survey were downloaded and analysed using an essentialist thematic analysis approach based on the guidelines developed by Braun and Clarke [37]. This approach was adopted as the overall aim of the study was to explore online experiences and therefore it represents a useful tool through which patterns and themes within the dataset can be identified and described. However, such an approach falls short of quantifying such patterns or themes and so a qualitative content analysis approach was not appropriate in this instance.

In order to undertake the inductive analysis at the semantic level the responses to the questions (see Table 1) were downloaded, read and re-read, with all interesting features of the data coded systematically into a coding framework. Themes were generated to form links between the separate codes and reviewed to check for consistency and coherence and final themes were produced upon further refinement. In undertaking the analysis, it was found that the data generated by the 50 respondents was sufficient to allow theoretical saturation to take place and for all the themes to be well formulated, thereby allowing our research questions to be fully addressed.

## Results

Thematic analysis revealed five themes reflecting empowering processes arising from members’ experiences within the group: *Connecting with others who understand*; *Accessing information and advice*; *Building confidence in interactions with health professionals*; *Facilitating treatment-related decision making*; *Improved adjustment and management*. In addition, two themes were identified which reflected disempowering aspects of their online experience: *Reading about the negative experiences of others* and *Feeling like an outsider*.

1 *Empowering processes within the PCOS online support group*

1.1 *Connecting with others who understand*

Many women reflected on how membership of the online group helped them realise that they were not alone, regardless of whether they actively posted messages or not:

The women appeared to benefit from reading about other members’ experiences and drawing comparisons between their own experience and the experiences shared by others. Some members appeared to make a downward social comparison as they considered their own symptoms were less severe than others. That said, members also compared themselves in a lateral fashion and described how simply knowing that others were experiencing similar problems was helpful in itself:

At the heart of the online experience appeared to be the feeling that members truly understood what it felt like to be living with PCOS. As one woman describes:

1.1 *Accessing information and advice*

Several women described how the online group was a useful source of information. It was common for members to know very little at the point of entry to the group:

Of particular benefit was information and advice about symptoms associated with the condition as well as future challenges they may present:

1.1 *Building confidence in interactions with health professionals*

Participants described how being a member of the online group helped them feel more able to seek help and communicate with health professionals about their condition. For example, one participant, who had lived with PCOS symptoms for over sixteen years and was awaiting test results of a possible diagnosis, recounted how having a better understanding of PCOS allowed her to feel more able to approach healthcare professionals for help:

By reading about the experiences of other members, some women were able to learn more about what treatments could be offered and this led them to become more confident in discussing treatment options with health professionals and on occasions challenging the recommendations provided. As one woman explained:

Indeed, many participants mentioned how they had encountered healthcare professionals who were unsupportive and lacked an understanding of PCOS. Thus, having an increased knowledge of PCOS appeared to help some participants feel more confident in their interactions with health professionals:

1.1 *Facilitating treatment-related decision making*

Treatment-related discussions were especially prominent within the messages exchanged between members. This provided the women with more information about options, both conventional and alternative, as well as helping them make decisions about their healthcare:

1.1 *Improved adjustment and management*

Many participants described how being a member of the group helped them feel more optimistic, hopeful and in control of their PCOS and therefore more able to manage the challenges associated with the condition. In particular, reading about how other women had addressed the challenges of living with PCOS appeared to be helpful:

2 *Disempowering processes within the PCOS online support group*

2.1 *Reading about the negative experiences of others*

Reading about the experiences of other women was something of a double-edged sword. On the one hand, it may help a member realise that things could be worse, but for some women it served to heighten their level of anxiety and worry about PCOS. As one member explains:

Another member described how they felt upset for other women with the condition who had shared less positive experiences:

For some women, reading through messages brought to their attention specific problems which they may face in the future and this led to greater worry:

Similarly, several women described problems associated with diagnosis and the management of symptoms. The impact of reading about such examples served to exacerbate feelings of hopelessness and reduce feelings of confidence in illness management by healthcare professionals:

Through participation in the group, some women felt that they had become too focussed on PCOS and this was not always helpful:

An awareness of the potential of the group to heighten worry about PCOS appeared to underpin the decision made by some women to limit their use of the group. As one woman explained:

2.1 *Feeling like an outsider*

Some members described difficulties in terms of joining in with conversations and engaging with other members:

Similarly, a lack of response to a message being posted or a response being unhelpful also appeared to contribute to feelings of being an outsider. As one woman explained:

*It has helped me deal with it on some occasions, because it can be a frustrating condition to have, and sometimes you just need to know someone else is having the same problems*. (P1)

*Although I do not post as regular as others, I enjoy reading other peoples experiences of similar situations, and also feel less isolated* [sic]. (P14)

*Feel less isolated and grateful that my symptoms aren’t as bad as for some*. (P39)

*I get strength from others who deal with it well. Some seem to be able to shrug it off. I can’t but I cope better knowing how many of us there are coping alongside each other. I don’t feel like such a freak*. (P42)

*It is fantastic to be able to discuss issues and concerns with people who completely understand what I am talking about, other that the support groups there is a lack of support as people cannot fully understand the condition unless they too suffer with it themselves* [sic]. (P14)

*I did not know anything really about PCOS and neither did the people giving me the diagnosis. I decided to go straight to those who’d have the best information and experience*. (P36)

*Yes i suffer with depression but could never really pinpoint why but now i know that its part of the condition so i am finding better ways to deal with it* [sic]. (P50)

*I felt desolate when I was first diagnosed. I’m yet to find out if my fertility is affected and all I can do is wait…I feel I have a better idea of what I’m up against now and I can’t describe the relief I feel at having people to talk to about this*. (P8)

*I feel more confident dealing with them and asking for help, as before I asked for help without knowing anything about the condition*. (P48)

*Knowing what things should be done and when from other posters allows you to be more assertive. I’ve felt that I had the information to challenge unfair treatment, particularly from my GPs surgery who don’t appear to understand PCOS*. (P64)

*I feel more empowered. I am usually significantly better informed on PCOS than the registrars I see when the consultant farms me out. I will never let another ‘specialist’ condescend to me and wave me on my way as happened in the past*. (P43)

*It heavily influenced my treatment choices as far as fertility was concerned. I chose natural methods to get fit and healthy after lots of medical intervention. Had I known about the group prior to IVF I think I may have tried fitness and lifestyle changes first.* (P34)

*I wouldn’t have tried laser unless someone on the discussion board had tried it first which they had and i have now had it done and it has made a massive difference to how I feel about me* [sic]. (P19)

*I felt desolate when I was first diagnosed. I’m yet to find out if my fertility is affected and all I can do is wait. The support group reassured me that this diagnosis doesn’t have to be the end of the world and I feel much happier knowing there are people out there who understand what I’m going through. I feel I have a better idea of what I’m up against now and I can’t describe the relief I feel at having people to talk to about this.* (P8)

*It has, it has made me want to fight it even more, I have currently lost 6 pounds in weight and I am wanting to lose more, I know that it will be a hard struggle but if it means beating pcos i will do it* [sic]. (P56)

*Makes you feel more anxious when you read about other people’s concerns and more severe problems – either you berate yourself for worrying about nothing or feel that the worst is yet to come.* (P37)

*Sometimes the sad situations others find themselves in is so heartbreaking it can impact on my own moods and emotional wellbeing – I deal with those situations by reviewing my own position and as clichéd as it may sound, by counting my blessings.* (P43)

*Occasionally it makes me worry about fertility as I have not tried for a baby yet and don’t want it to be really difficult when I do.* (P35)

…*you can end up feeling depressed by the fact so many people have the same problems getting diagnosed and then getting help*. (P59)

*I have found that very few people have been helped by the treatments offered, and that the help we want (for example laser treatment on the NHS) is not available*. (P30)

*It can be easy for it to become your life and determine who you are rather than getting on with life and it being one aspect of you. Alot of people use forums to wallow in the issue* [sic]. (P18)

*Everyone can appear to be in a desperate state maybe only people in dire need regularly access? Dealt with this by limiting use as it was making me more depressed and feeling hopeless* [sic]. (P25)

*Sometimes there are cliques and strong friendships formed and you can’t infiltrate them*. (P36)

*I don’t always want to encroach on a group that seems established and bonded*. (P28)

*I tried it once and the communication is very odd – sometimes unreciprocated, or mostly people are posting about their own concerns but not really responding in a useful way (if at all) to other people – particularly since they mostly are not healthcare professionals*. (P37)

## Discussion

The aim of this study was to explore the experiences of women who access an OSG for Polycystic Ovary Syndrome. Whilst there has been a growing body of literature examining the role of OSGs for people living with chronic illness, there has been a notable lack of work which has considered this specific condition. The present study therefore represents a unique contribution to our understanding of how OSGs may help women living with this challenging condition.

Our qualitative analysis revealed a range of positive benefits arising from OSG use. First of all, through accessing a PCOS-OSG, members appeared to derive benefit from realising that they were not alone. In addition, members were able to compare themselves with regards to a range of illness attributes, such as symptom severity, as they read through the messages posted to the group. Both these benefits may be explained with reference to social comparison processes, which have similarly been identified in other chronic illness OSGs [24,29]. Through lateral comparison, members may realise that they are not alone or unique in their suffering, but that many others may share similar experiences. Similarly, by reading about the symptoms experienced by other members and realising that they are more severe than one’s own may lead to a downward social comparison. Through downward social comparison, members may come to appreciate that things could be worse and this has been shown to be a commonly reported benefit of OSGs [33]. Nevertheless, it must be noted that downward comparisons can also elicit negative feelings such as anxiety and fear in some members who worry that their symptoms may one day worsen, as well as sadness on behalf of the perceived suffering of others.

Membership of the OSG also carried benefits with regards access to information and advice, particularly for those who lacked understanding of the condition. This finding is consistent with the extant literature which has demonstrated that one of the key benefits to patients arising from OSG membership is access to information, advice and support [22]. It would appear then that in specific instance of PCOS, those women who access the OSG may have access to more helpful information which may not always be forthcoming from health professionals. Indeed, through this information acquisition, many women also reported feeling more confident to discuss the condition, its symptoms and particularly treatment options with their health professionals and this was seen as especially helpful where there was a lack of understanding or disagreement over treatment recommendations. More generally, membership appeared to be helpful in contributing to an improved ability to manage the condition and helped to reduce feelings of loneliness and foster a sense of control and hope for the future.

Recent work in the field of HIV/AIDS suggests that participation in OSGs may help individuals realise they are not alone which in turn contributes to lower levels of depression and a greater sense of optimism and perceived control over their illness [31]. Future research needs to explore using more methodologically robust study designs the impact of OSG membership on a range of psychosocial measures as well as identifying the causal mechanisms involved.

Despite the various positive aspects of OSGs described by members, two problematic features were also identified. The first of these, relates to the potential harm which may arise through reading messages posted by other members describing negative illness-related experiences. Whilst reading about the experiences of others was previously noted as a potential benefit, it would appear that it may also have a potentially negative impact on some members by bringing to their attention or making more salient, aspects of their condition which they either hadn’t or didn’t wish to consider at that point in time. The impact of reading about negative experiences has yet to be fully explored within the literature but it is conceivable that some group members may become anxious or worry more about their condition as a result. Similarly, it may also impact on how members use the OSG. As noted in our findings, some members altered how they engaged with the group through accessing it less often but it is also conceivable that some members may have discontinued their use of the group. This issue merits further research attention in order to fully assess how reading member messages may negatively impact on patient wellbeing, engagement and membership of online groups. Future research may usefully seek to identify and understand the patient-related factors as well as message-related factors which cause such a situation to arise. This will help develop more helpful guidance to patients on how to successfully engage and benefit from OSG membership, as well as provide guidelines on how OSGs can be managed and moderated to maximise benefits to its users.

The second area of concern for group members relates to factors which may lead an individual to feel like an outsider. According to our analysis, this may manifest itself through perceived group dynamics and bonds between certain members or may simply be through the absence of a response to a message posted. In either event, some group members felt that they didn’t belong or that their efforts were not reciprocated. The existence of this potential limitation may be dealt with through a variety of ways but is almost certainly likely to include the role of the group moderator. As moderators, often patients themselves, volunteer to facilitate the group and interaction between members, it may be that they can play a more active role in helping new members integrate into a group or ensuring that messages posted receive some form of reply. That said, current research suggests that moderators may well be stretched with regards time available to effectively facilitate OSGs, especially where there are sizeable numbers of members frequently posting [38].

A number of limitations in this study must be acknowledged when considering the findings. First of all, we sampled from only one UK based OSG supporting women with PCOS and therefore it is conceivable that other groups may function differently or have different dynamics which may have an impact on how members experience them. Furthermore, our qualitative study focussed on a comparatively small number of women and whilst this study is one of the first dealing with PCOS, it may benefit from a much larger sample size across multiple groups and in-depth interviews, in order to be confident about generalising the findings. Moreover, like much research in this area, we do not know about those members who ceased to use the group. Whilst we did include participants who varied not only in terms of how long they had been using the group but how often they did so, we failed to capture the views of those who no longer participated. It may be the case that we therefore obtained the views of members who, on the whole, were relatively happy with their online experience but an altogether different picture may emerge if non-active members were examined. Finally, we must acknowledge the fact that bias may exist with regards the sample of women who chose to engage with our study. It is entirely plausible that the women who responded to our research request were already pro-active with regards their health and motivated to seek help and support. Therefore, our results must be considered in light of any bias that may be present. Looking to the future, further research is needed to develop this area in more depth and in particular to confirm and extend the findings of the present study by engaging in a quantitative survey using a larger sample of women living with PCOS, and asking questions based on the findings of the current study. Furthermore, it would be useful to explore reasons for non-engagement with online support groups as well as comparing the support provided and benefits derived from both online and offline support groups.

## Conclusions

For women living with PCOS, participation within an online support group appears to offer various benefits through interacting with other sufferers. In particular, the opportunity to share and discuss their own experiences may be helpful in terms of learning to manage and cope with their condition. However, the online support group is not without its limitations and there is the risk that some women who engage with the online support group may suffer through reading about other women’s distress or indeed feel more isolated.

Overall, the findings of this work suggest that for some women, the online support group is a viable alternative to traditional offline forms of social support and health professionals may wish to discuss with individual patients whether it represents a useful medium through which to connect with other women facing similar challenges. The online support group may represent a relatively inexpensive and convenient form of support that may address an important gap in current support provision for this group of patients. Furthermore, it may also provide a venue through which partners and family members may come together to share their experiences and benefit from support as they too are likely to be impacted upon by this condition.

## Competing interests

The authors declare that they have no competing interests.

## Authors’ contributions

This study was carried out as part of an MSc research project at the University of Nottingham by SH. NC supervised the project and both authors drafted and revised the manuscript. Both authors read and approved the final manuscript.

## Pre-publication history

The pre-publication history for this paper can be accessed here:

http://www.biomedcentral.com/1472-6874/13/51/prepub
